# What's Up With Everyone? A qualitative study on young people's perceptions of cocreated online animations to promote mental health literacy

**DOI:** 10.1111/hex.13507

**Published:** 2022-05-04

**Authors:** Sachiyo Ito‐Jaeger, Elvira Perez Vallejos, Thomas Curran, Paul Crawford

**Affiliations:** ^1^ School of Health Sciences, Faculty of Medicine and Health Sciences The University of Nottingham Nottingham UK; ^2^ NIHR Nottingham Biomedical Research Centre Nottingham UK; ^3^ School of Medicine Faculty of Medicine & Health Sciences Nottingham UK; ^4^ Department of Psychological and Behavioural Science The London School of Economics and Political Science London UK

**Keywords:** animation, cocreation, film, mental health literacy, online, young people

## Abstract

**Introduction:**

Adolescence and young adulthood are especially critical times to learn about mental health, given that 75% of mental health issues are developed by the age of 24. Animations have great potential to effectively deliver mental health information to young people. A series of five short animated films to promote mental health literacy were created with and for young people in partnership with the multi‐award‐winning independent animation studio, Aardman Animations. The aim of this study was to explore young people's perceptions of the cocreated animated films.

**Methods:**

Seven Youth Juries were conducted to capture young people's opinions and recommendations about the content related to mental health literacy and presentation style of the cocreated animated films. Thematic analysis was used to analyse the audio transcripts.

**Results:**

Many participants reported a view that the animated films had the potential to promote mental health literacy, especially for understanding mental health and reducing stigma. Some recommendations were provided to improve the films, such as including subtitles and having a better transition to the companion website.

**Conclusion:**

Cocreated animations have great potential to promote the mental health literacy of young people. We hope that the findings from the present study will inform future media development to make them as effective as possible.

**Patient or Public Contribution:**

Young people were actively involved in the development, production, implementation and evaluation (up to the time before data analysis) of the animated films.

## 1 INTRODUCTION

The growing burden of mental ill‐health facing society is clear. Increases in mental disorders observed by the Lancet Commission on global mental health, for instance, are estimated to cost the global economy $16 trillion by 2030.^1^ Young people are particularly at risk. Transitioning into adulthood is challenging as young people face pressures and issues related to mental health, such as perfectionism, independence, loneliness, social media and competitiveness.^2^, ^3^, ^4^, ^5^, ^6^ Adolescence and young adulthood are especially critical times to learn about mental health, given that 75% of mental health issues are developed by the age of 24.^7^ An objective of learning about mental health is to enhance mental health literacy.^8^

Mental health literacy is defined as ‘understanding how to obtain and maintain positive mental health; understanding mental disorders and their treatments; decreasing stigma related to mental disorders; and, enhancing help‐seeking efficacy (knowing when and where to seek help and developing competencies designed to improve one's mental health care and self‐management capabilities)’.^9^ Interventions oriented towards the improvement of mental health literacy have tremendous potential in addressing deteriorating youth mental health.

### 1.1 Digital media

Creative practices, in particular, have documented potential for transforming people's mental health literacy.^10^, ^11^ One of the methods used to creatively promote mental health literacy is digital media. The use of online and social networks is especially suited to reach young people because the modality of delivery reflects how they are consuming information. The vast majority of young people aged 16–24 years in high‐income countries use social media.^12^ In the United Kingdom, 99% of young people have a smartphone and 92.7% of young people use social networking.^12^, ^13^ Not surprisingly, social media‐friendly digital films are one of the most effective and cost‐effective media to improve mental health literacy among young people.^14^, ^15^

### 1.2 Cocreation of digital films

To maximize the impact on the target audience, it is suggested that media content should be cocreated with end‐users at the centre of the creative process.^16^ In academic parlance, cocreation is defined as ‘the collaborative generation of knowledge by academics working alongside stakeholders from other sectors’.^17^ Stakeholder engagement generates inclusive and representative conversations among multiple sectors that can drive the development of meaningful and more relatable media interventions.^18^ Therefore, engaging end‐users is crucial when creating a digital film intervention. However, according to a recent scoping review, only a few of the mental health film interventions were cocreated with end‐users.^14^ Of these, the authors argue that animations are perhaps one of the most useful media for cocreation since they contain an active voice, while enabling anonymity for those end‐users involved in the creative process.^14^, ^19^

### 1.3 Animation

Animation has great potential to communicate knowledge and promote learning.^20^ Animation has been used to communicate health‐related information and has been found to be effective in improving health literacy and intentions to change health‐related behaviours positively.^21^, ^22^, ^23^ The recent study by Dunn et al.^19^ is the first of its kind to report on a cocreation process, in which young people were involved in creating animations to communicate mental health information.^19^ In this study, a group of young people who had been diagnosed with depression created a short animation about depression and therapy with filmmakers. The film was promoted on social media, and was viewed 12,000 times on YouTube. In another recent study, young people cocreated a series of short animations to raise awareness of mental health issues commonly experienced by young people.^24^ The cocreated animations gained 16,000 views on social media in the 12 months following its launch.

These previous studies show that animations have great potential to deliver mental health information to a large number of young people. However, no studies have involved young people in creating animations to promote mental health literacy throughout the creative process (i.e., development, production, implementation and evaluation) as suggested by Jirotka et al.^16^ In the present project, young people were actively involved in the cocreation process from the development to the evaluation (up to the time before data analysis). Furthermore, the present study is the first to invite an independent group of young people (i.e., different from the young people who were involved in creating the animations) to evaluate the cocreated mental health animations. The use of an independent sample is an important practice as it helps to reduce bias.

### 1.4 The What's Up With Everyone project

The What's Up With Everyone (WUWE) project is a campaign developed to promote mental health literacy among young people. In this project, a series of five short animated films were created with and for young people in partnership with the multi‐award‐winning independent animation studio, Aardman Animations. Each film is 40–50 s long and focused on an issue related to loneliness and isolation, perfectionism, competitiveness, social media and independence. The films are embedded in a companion website^25^ and are also available on Aardman's YouTube Channel,^26^ Instagram,^27^ Twitter,^28^ Facebook^29^ and TikTok.^30^ Full details of the project and context can be accessed on the website and the UKRI announcement.^31^ In a 4‐month media campaign following the launch of WUWE on 8 February 2021, the films reached over 17 m people, with Instagram the highest performer, followed by Twitter and Facebook. By 4 June 2021, there were 4.93k followers, with more than 638k views of the films across the channels. In this initial period, the companion website alone attracted 33.1k users with 44k sessions, 101k unique page views, 994k total page views and 4.5k returning users.

#### 1.4.1 Cocreation with young people

As recommended by Jirotka et al.,^16^ the end‐users (i.e., young people) were actively involved in the development, production, implementation and evaluation of the animated films, by participating in workshops. In the early development stage, a group of nine young people identified five key issues related to mental health in the population: perfectionism, independence, loneliness and isolation, social media and competitiveness. These five issues are aligned to the issues and pressures that young people currently face.^2^, ^3^, ^4^, ^5^, ^6^ An additional group of 40 young people confirmed the significance of the five issues. This group of young people was further involved in the idea generation and character and environment build. Each workshop at this stage consisted of young people, the production team (a director, a creative director, and a producer) at Aardman Animations and a clinical professional (e.g., clinical psychologist).

For the production stage, 5 of the 40 young people were selected to be in the inner group based on the level of engagement during the development stage while ensuring a diverse group (i.e., gender, ethnicity). The inner group met with the production team more frequently and provided more in‐depth feedback to the team than the outer group (i.e., 35 young people). Both groups were involved in the script and animatic development, whereas the inner group gave feedback on the final film production. The scripts evolved based on the cocreators' feedback, producing 18–21 versions for each theme. Each of the five main characters was voiced by the young people rather than actors. In the implementation stage, the inner group gave suggestions on the public relations strategy, for example, which influencer would be a good fit for the media campaign.

The current paper reports the methods and results of the final stage, the evaluation stage, focusing on the qualitative data. The quantitative data of the same project, collected via online questionnaires, have been reported in Curran et al.,^32^ in which the data revealed an increased level of mental health literacy (e.g., willingness to seek help) after watching the animations.

### 1.5 Objective

This study aims to explore young people's perceptions of cocreated animated films. We adopted a qualitative approach and facilitated a series of discussions (i.e., Youth Juries [YJ]) among young people to capture their opinions and recommendations about the content related to mental health literacy and presentation style of the animations, to share the knowledge for future research and animation development.

## 2 METHODS

### 2.1 Participants

Following the approval of the study by the research ethics committee at the University of Nottingham, an independent sample of young people was recruited solely for the purpose of evaluating the animations by distributing flyers and posting the information on an online recruitment site. The flyers, which contained the description of the study and the eligibility for participation, were distributed via email to mental health organizations, high schools, colleges and universities in the United Kingdom. The eligibility for participation included (1) English speaker living in the United Kingdom, (2) age 17–21 years at the time of recruitment and (3) with access to the internet and a computer, smartphone or tablet. Potential participants were asked to submit the Expression of Interest form. Thirty‐nine (mean = 19.46. SD = 1.57) young people participated in YJ. The demographic information is shown in Table 1.

**Table 1:** Demographics of the participants

		*N*	%
Gender			
Female		31	79.5%
Male		8	20.5%
Age			
17		7	17.9%
18		5	12.8%
19		5	12.8%
20		8	20.5%
21		13	33.3%
22		1	2.6%
Ethnic background			
White: English/Welsh/Scottish/Northern Irish/British		19	48.7%
White: Irish		1	2.6%
White: any other background		3	7.7%
Multiple ethnic groups: White and Black African		1	2.6%
Multiple ethnic groups: White and Asian		1	2.6%
Asian: Indian		5	12.8%
Asian: Pakistani		3	7.7%
Asian: Bangladeshi		3	7.7%
Asian: Any other Asian background		2	5.1%
Prefer not to say		1	2.6%
Religion			
None		20	51.3%
Christian		5	12.8%
Hindu		3	7.7%
Muslim		6	15.4%
Sikh		1	2.6%
Prefer not to say		3	7.7%
Other		1	2.6%
Highest level of qualification^a^ (participant)			
Level 1^b^		1	2.6%
Level 2^c^		9	23.1%
Level 3^d^		17	43.6%
Level 4 or above^e^		4	10.3%
Other qualifications^f^		7	17.9%
Prefer not to say		1	2.6%
Highest level of qualification^a^ (parent/guardian/carer)			
Level 1^b^		2	5.1%
Level 2^c^		3	7.7%
Level 3^d^		3	7.7%
Level 4 or above^e^		13	33.3%
Other qualifications^f^		12	30.8%
Prefer not to say		6	15.4%

^a^ Qualification levels (UK census).

^b^ 1–4 O levels/CSEs/GCSEs(any grades), Entry Level, Foundation Diploma, NVQ Level 1, Foundation GNVQ, Basic Skills.

^c^ 5+ O levels (passes)/CSEs (Grade 1)/GCSEs (Grades A*–C), School Certificate, 1 A level/2–3 AS levels/VCEs, Higher Diploma, NVQ Level 2, Intermediate GNVQ, City and Guilds Craft, BTEC First/General Diploma, RSA Diploma.

^d^ 2+ A levels/VCEs, 4+ AS levels, Higher School Certificate, Progression/Advanced Diploma, NVQ Level 3, Advanced GNVQ, City and Guilds Advanced Craft, ONC, OND, BTEC National, RSA Advanced Diploma.

^e^ Degree (e.g., BA, BSc), Higher Degree (e.g., MA, PhD, PGCE), NVQ Level 4‐5, HNC, HND, RSA Higher Diploma, BTEC Higher level, Professional qualifications (e.g., teaching, nursing, accountancy).

^f^ Other vocational/work‐related qualifications, Foreign qualifications.

### 2.2 Procedure

Young people who confirmed their willingness to participate in the study received and submitted the consent form and a demographic questionnaire via email. The consent form included a consent for audio and video recording, but participants were reassured that they did not need to turn on their camera if they did not feel comfortable. One week before the study, participants received an email containing the links to the five films and were asked to watch them before the session.

YJ, which are similar to focus groups, have an explicit objective of arriving at clear recommendations.^33^ YJ were moderated by two of the authors who specialize in psychology and digital technologies. One of the moderators had substantial experience in facilitating Youth Jury sessions. The Youth Jury started with an ice breaker exercise, followed by a description of the Youth Jury methodology and the aims of the study. Participants were asked to become jurors and to discuss what they think about the evidence presented (i.e., the animated films) with the whole group. The moderators explained the importance of the jurors' input as it will be used for future media development to promote mental health information to young people.

After watching each of the five animations in a randomized order, the moderators first asked participants a broad question, such as ‘Can you explain what you thought about the animation?’ We then asked more specific questions related to each component of mental health literacy, such as ‘What about the animation did you find useful or not useful for your understanding of mental health and loneliness? Did any expressions or lines stand out to you?’ After watching all five films, participants were then asked to share their thoughts on the anthology of films overall. We introduced the concept of mental health literacy at the end of the focus group. Each session lasted for one and a half hours and was audio‐recorded and transcribed verbatim. Participants were compensated financially (£20) for their participation. A total of seven YJ were conducted, and each session consisted of five or six participants. Data saturation became apparent, with major trends clear by the end of the seven YJ; thus, an eighth would not have been appropriate. All YJ were conducted online via Zoom as the United Kingdom was under Covid‐19 restrictions at the time of the study.

### 2.3 Data analysis

Thematic analysis was used to analyse the transcripts.^34^ Thematic analysis has been used to understand young people's opinions and recommendations about a newly developed digital intervention.^35^ Following Braun and Clarke's^34^ six phases of thematic analysis, one of the authors (1) became familiar with the content by reading and rereading the transcripts, (2) generated initial codes, (3) searched for themes, (4) reviewed the themes, (5) defined and named themes and later (6) produced the report. Throughout the process, research meetings were held for debriefing and discussing emergent themes among the authors.

## 3 RESULTS

**Figure 1:**
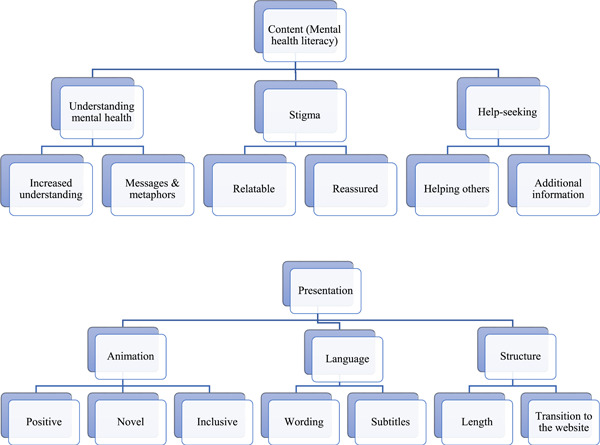
Themes related to the content and presentation of the WUWE animations. WUME, What's Up With Everyone

The YJ discussions focused on the content of the animation, particularly related to the three components of mental health literacy (i.e., understanding mental health, reducing stigma, help‐seeking efficacy), and the presentation style of the animations. As presented in Figure 1, two themes were generated for each component of mental health literacy, while three themes housing seven subthemes were generated for the presentation style of the films.

### 3.1 Content

Many participants reported a belief that the animated films had the potential to promote mental health literacy, especially for understanding mental health and reducing stigma. Themes concerning each component of mental health literacy are described below.

#### 3.1.1 Understanding mental health

##### 3.1.1.1 Increased understanding of mental health issues

A majority of participants reported a view that the animated films had the potential to increase young people's understanding of mental health issues. They stated that the short films made them realize for the first time that some of the issues presented within the animations are related to mental health.

Participant 16: People may see [perfectionists] as like annoying, trying to get everything perfect and to see them like trying hard but I think it is good that it shows they don't think… like one little thing goes wrong and they just spiral downwards and it is good like it shows like it is just as dangerous as other mental health issues, rather than you know just being like stuck up or something. (YJ3: Perfectionism)

##### 3.1.1.2 Messages and metaphors

Some messages used in the animations were perceived to be helpful to understand the issues. The messages found to be helpful included ‘You are not perfect, and that's ok’ (perfectionism), ‘And who knows? Maybe they know the feeling too’ (independence), ‘Remember who you are running for’ (competitiveness) and ‘So you do you, Daisy, and I'll concentrate on me’ (social media).

Participant 8: The phrase in it was ‘who are you running for?’… I think being competitive against yourself is more important because you can see the changes more and it is not as toxic. I think being competitive against others to a certain extent, it is all right if like oh I want to win this race or I want to be better in this… but if it is like really everything it gets really toxic and really harmful. (YJ2: Competitiveness)

Additionally, some metaphors, such as the bin in the Perfectionism film and the thorns in the Social Media video, were believed to enable young people's understanding of mental health.

Participant 10: I think the thorns that do obviously seem to grow erm do very much represent a way in a world you can find yourself on social media. I think it is very important that especially when she stuck her head out, that when you engage erm with social media I think it is important that you take a… it is almost like a third person view step back to greater understand the mass of what is happening and the situation. Erm so I think it very much found a way to represent how to react to negative erm sort of situations on social media without directly including sort of negative things. You can kind of gather the thorns were the bad things, but it didn't give you any specifics and then in an analogy to see that stepping away and rising above it gives you the sort of mental position and state to be able to react more positively to it. (YJ2: Social Media)

#### 3.1.2 Stigma

Many participants believed that the animated films would help to reduce stigma related to mental health issues because they are relatable to young people, and they reassure young people that other people have experienced similar issues.

##### 3.1.2.1 Relatable

The majority of the participants reported finding the animations very relatable to their lives and experiences. They reported a belief that because of this relatability, the animations had the potential to reduce stigma related to mental health issues. It was mentioned that other films that they had watched in the past were not as relatable as the WUWE material. The comical style of the animations appeared to contribute to the feeling of being relatable.

Participant 25: If it was too serious then maybe the message wouldn't be as effective because for example if you were a person who was like a perfectionist and like this, you might not see it as being like a completely debilitating problem erm but by having it in the slightly light, funny style, you can maybe see it as more relatable. Whereas, if it was all doom and gloom you would be like this isn't really me. (YJ5: Perfectionism)

##### 3.1.2.2 ‘Normalizing’ the issues

Many participants reported that the animated films made them realize that other people struggle with the same issues. They explained that ‘normalizing’ the issues would help reduce the stigma for young people.

Participant 11: People who have stigma towards mental health, I think they separate themselves as one like group of people and then the mental health people in another group, another category but this animation kind of shows that everybody has erm… everybody worries and stresses and it kind of makes it less erm of like a new concept, something that they can never experience. That kind of eases them in to knowing that they are able to worry…. (YJ2: Independence)

#### 3.1.3 Help‐seeking efficacy

The animations prompted discussions about helping people with mental health issues. However, participants described that more information is necessary for them to learn about seeking help.

##### 3.1.3.1 Helping people with mental health issues

Some participants indicated that the animations made them realize the difficulty of identifying people with mental health issues because some people may conceal their emotions. To address the problem, it was suggested that young people could reach out to people by showing small gestures (e.g., saying hi) as one of the characters in the Loneliness video did.

Participant 1: It is not always clear to see when someone is going through that which is why maybe you should erm try your best to just ask, even if it is just casually, just like are you OK and kind of the powerful effect that can have for someone who is not erm able to talk about it so openly. (YJ1: Loneliness)

##### 3.1.3.2 The necessity of more information on help‐seeking

Many participants, however, reported that the films did not contain enough information about help‐seeking. The animated videos are linked to and embedded in a companion website,^25^ which contains further information on help‐seeking, including a list of organizations that offer help to people with mental health concerns. Participants stated that reading the website would help people learn more about help‐seeking. As discussed in the ‘transition to the WUWE website’ section below, it is therefore crucial that the effective transition from the films to the companion website is in place.

Participant 17: I think the video itself would definitely like help you understand it and reduce stigma and make you think it is more normal, but to help like, help seeking, having the text, especially at the end saying like with the links to the website and stuff, that is going to help people seek mental health help. (YJ3)

### 3.2 Presentation

#### 3.2.1 Animation

##### 3.2.1.1 Positive: Interesting and comforting

Positive feedback was provided on the animation style in general. Most participants reported that these animated films are interesting, entertaining, playful and comforting.

Participant 1: I just wanted to say that I really like the idea of these videos as a whole. … they are really engaging, and it is really interesting content erm that I think should be kind of more common, like we should see it much more often. (YJ1)

Participant 20: I really liked the comforting tone of it. I think at times like that you can be quite sort of stressed, and it was a nice comforting tone to reassure you. (YJ4: Social Media)

##### 3.2.1.2 Novel and refreshing

Some participants expressed that although they have watched many mental health‐related videos growing up (e.g., at schools), the WUWE videos are unique and refreshing to watch, and thus they pay more attention. They reported that these films have high production quality and are more detailed than others, and also animation is used less often to portray mental health issues than live action.

Participant 1: …it was kind of different from like little clips that I have seen before… it was quite detailed in terms of like what exactly [the characters] were thinking or exactly they were going through at the time. (YJ1: Perfectionism)

Participant 31: I have had an overdose of real people, erm I think animation is refreshing, it makes me relook at the topic. I think I have just seen too many mental health, public information, public health things that are physical people, and the message is nullified. With the animation it is making me look at it with fresh eyes again. I pay more attention. (YJ6)

##### 3.2.1.3 Inclusive

Some participants reported that they enjoyed the inclusiveness shown in the animations. They stated that the animated characters, especially animal characters, allow anyone, regardless of ethnicity, to relate to the characters. Participants also appreciated including multiple accents as it shows diversity and inclusivity.

Participant 33: If it is real people… even if it is unconscious, it is slightly harder to see yourself in their shoes just because I don't know they might look different or you know different race or a different gender whereas if you have it as like an animated character, it is kind of anonymous in its own right and anybody can kind of relate to it, so I think having it as an animation definitely helps explain it and make you feel like you know you know that feeling so it is not as erm… it is not as cold, it is a nice… it just makes the whole experience a lot warmer when you watch it. (YJ6)

Participant 30: I do like the diversity and the more that yes you have included more than one accent I think that is nice to hear. (YJ6)

#### 3.2.2 Language

##### 3.2.2.1 Wording

Some recommendations were provided for the wording used in the animation. In particular, many participants stated that the phrase ‘be confident’ (independence) is not helpful and suggested using the alternative ‘it's okay to ask for help’ as more effective and best suited for this particular film.

Participant 13: I think maybe like at the end it says be confident, maybe instead tying in that thing of like asking for help because I feel that is more the kind of message behind it [YJ3: Independence].

##### 3.2.2.2 Subtitles

Some participants proposed including subtitles in the short films. They stated that young people use subtitles when they watch movies to help them better hear and understand the content. Subtitles are especially useful to understand diverse accents in the United Kingdom. Although an option to turn on subtitles is available on YouTube, participants recommended that subtitles should be the default for the films for all social networks.

Participant 32: … I think a lot of people just like subtitles anyway, and I know I do just makes things easier to hear. (YJ6)

#### 3.2.3 Structure

##### 3.2.3.1 Length

Most participants stated that the length of each film was appropriate to deliver the message effectively, enhancing attention.

Participant 36: I liked that it was short so it doesn't feel really overwhelming so it encourages people to think well you know if other people can talk about it in small sections, then I can start to deal with it and it is not something that is just like all consuming, it is easier to process. (YJ7)

##### 3.2.3.2 Transition to the WUWE website

Some participants stated that they accessed the companion website because its address was included at the end of each film. Other participants, however, recommended making some changes to the videos to increase traffic to the website. Proposals included adding the logos of the universities and mental health organizations in the films to increase the credibility of the information on the website.

Participant 25: If it did have like at the end on that screen like university of whatever then I would see it more as like maybe something more that could actually help me because it is something that is like proven. (YJ5)

The transition to the website is important as the website contains additional information about how each of the life challenges addressed in the films can impact on mental health, approaches to self‐help and direction on seeking help. As discussed in the ‘help‐seeking’ section above, accessing the website is crucial as participants reported that the films do not provide enough information on help‐seeking.

## 4 DISCUSSION

In the present study, we captured young people's opinions and recommendations about the content related to mental health literacy and presentation style of the animated films by facilitating a series of YJ. Many participants believed that watching the animated films had the potential to improve young people's mental health literacy, especially for understanding mental health and reducing stigma. Additionally, participants provided positive feedback and some recommendations for the presentation style of the animations.

Most notably, many participants believed that the animations had the potential to reduce stigma because they are relatable and reassure young people that others have experienced similar challenges. This is particularly noteworthy, given that these animations are cocreated with young people to develop animations that are relatable to other young people. Cocreation is an essential practice that drives the development of meaningful and more relatable media intervention and maximizes impact on the target population.^16^, ^18^ Stakeholder engagement is one of the pillars for responsible research and innovation and a key element for developing new media interventions in a socially desirable and acceptable way.^16^ The current results demonstrate and reconfirm the importance of cocreation with end‐users.

In terms of understanding mental health, participants considered the metaphors in the films helpful. Metaphors are useful to understand abstract, complex phenomena.^36^ Therefore, they have been actively used to describe mental illness. Metaphors in portraying mental health issues such as depression have been featured previously in animations.^37^, ^38^, ^39^

Although some participants reported that the films addressed one important aspect of help‐seeking efficacy, helping others,^40^ many participants expressed that the animations included only limited information on seeking help. However, participants reported that the animations contributed to their understanding of mental health issues and reducing stigma related to mental health issues, and past research shows that the limited knowledge of mental health and stigma are two of the prominent barriers for people to seek help.^41^ As such, watching the animations alone may already encourage some young people to seek support. Nonetheless, for young people who require additional information on seeking help to cope with life challenges as young people see them (e.g., mental health organizations for young people), it is vital to have a better transition from the films to the companion website. One of the proposals made to enhance the transition was to add the logos of the universities and mental health organizations in the animated videos to increase the credibility of the information on the website. Previous studies show that the creator's credibility positively influences users' trust in digital health resources,^42^, ^43^, ^44^ and trust is one of the key factors for deciding whether to use a particular kind of technology.^45^ Thus, future media creators should ensure that the involvement of the established institutions is clearly presented in the media outlet.

In general, positive feedback was provided on the presentation style of the animations. The high production quality was achieved by a partnership with a multi‐award‐winning animation studio. It has been discussed that there is a need to attend to the production quality so that the digital video intervention is as effective as possible.^14^ The present study highlights the importance of interdisciplinary collaborations with creative industrial partners.

In addition, participants perceived the films as inclusive in their use of diverse accents and animal characters relatable to a diverse population. Inclusiveness is vital for mental health promotion, given that minority groups, including Black, Asian and minority ethnic (BAME), are portrayed as a ‘hard to reach’ population in primary care mental health services.^46^ Although other factors, such as political climate, are barriers for minority groups to access mental health services, it is hoped that these inclusive videos provide comfort for as diverse a population as possible and encourage help‐seeking.^46^

Subtitles were recommended to be included in the films. Past research shows that subtitles are beneficial for everyone as they increase understanding of, attention to and memory for the material.^47^ Subtitles are important to further improve the inclusiveness of the films as they benefit people who are deaf or hard of hearing and people whose native language is not English.^47^ Subtitles are also helpful for native English speakers who are not hard of hearing, especially when diverse accents are used in the films.

### 4.1 Limitations

Several limitations of the study should be noted. First, the majority of participants are female. Although we aimed to recruit more male participants by exclusively recruiting them on an online recruitment website, we received only limited interest from males. Second, we conducted YJ online due to the Covid restrictions during the study. This might have excluded young people with limited resources as only those with a digital device and internet access or enough mobile data were able to participate. Although 99% of young people aged between 16 and 24 years in the United Kingdom have a smartphone,^13^ limited mobile data or internet access might have prevented some young people from participating in the study. To include young people with limited resources, future studies should be conducted both in person and online.

## 5 CONCLUSION

This study provided insight into the content and presentation style of cocreated online animated films to promote mental health literacy. The results indicate that cocreated animations have the potential to promote the mental health literacy of young people. However, several recommendations were also made to improve the films. We hope that the findings from the present study will inform future media development to make them as relatable and effective as possible.

## CONFLICTS OF INTEREST

The authors declare no conflicts of interest.

## Data Availability

The data that support the findings of this study are available from the corresponding author upon reasonable request and with Research Ethics Board approval.
